# Infrarenal aortic reconstruction using autologous femoral vein for a mycotic aneurysm caused by *Streptococcus equi*

**DOI:** 10.1093/jscr/rjab388

**Published:** 2021-09-14

**Authors:** Paul Ghaly, Delfino Di Mascio, Mauro Vicaretti

**Affiliations:** Vascular Surgery Department, Westmead Hospital, WSLHD, NSW, Australia; Vascular Surgery Department, Westmead Hospital, WSLHD, NSW, Australia; Vascular Surgery Department, Westmead Hospital, WSLHD, NSW, Australia

## Abstract

A common source of infection in equine, *Streptococcus equi*, is an uncommon pathogen in humans, rarely identified as the cause for mycotic aortic aneurysms. Typically associated with consumption of unpasteurized milk or contact with horses, *S. equi* can result in severe bacteremia, endocarditis and meningitis. We describe the presentation and successful management of a 69-year-old retired equestrian who underwent infrarenal aortic resection and reconstruction using autologous right femoral vein for a *S. equi* mycotic aneurysm.

## INTRODUCTION

Used by Osler in 1885 to describe infected aneurysms due to bacterial endocarditis, the term ‘mycotic aneurysms’ encompasses a life-threatening condition involving infection of pre-existing arterial aneurysms or development of new aneurysms because of infectious arteritis [1, 2]. Infected aneurysms are uncommon, usually caused by bacterial endocarditis, bacteremia and trauma. *Staphylococcus aureus*, *Salmonella species,* Tuberculosis, mycobacterium, *Streptococcus pneumoniae* and fungi are all causative organisms [1, 3, 4]. *S. equi* is a rare cause of mycotic aneurysms with a recent review identifying 12 cases in the literature [5]. We report a case of an infrarenal reconstruction using right femoral vein for management of a *S. equi* mycotic aneurysm in a retired hobby equestrian.

## CASE REPORT

A 69-year-old retired male with a background history of ulcerative colitis (UC) and primary sclerosing cholangitis (PSC) presented with a 3-week history of generalized malaise, fevers, abdominal and back pain. He denied any diarrhoea, melaena, nausea or vomiting, recent travel or sick contacts. He had no surgical or family history of aortic aneurysms. Since retirement, his hobbies included farming with regular contact with horses and show ponies. He was a non-smoker and denied any intravenous (IV) drug use. A full blood panel demonstrated elevated inflammatory markers and deranged liver function tests.

**Figure 1:**
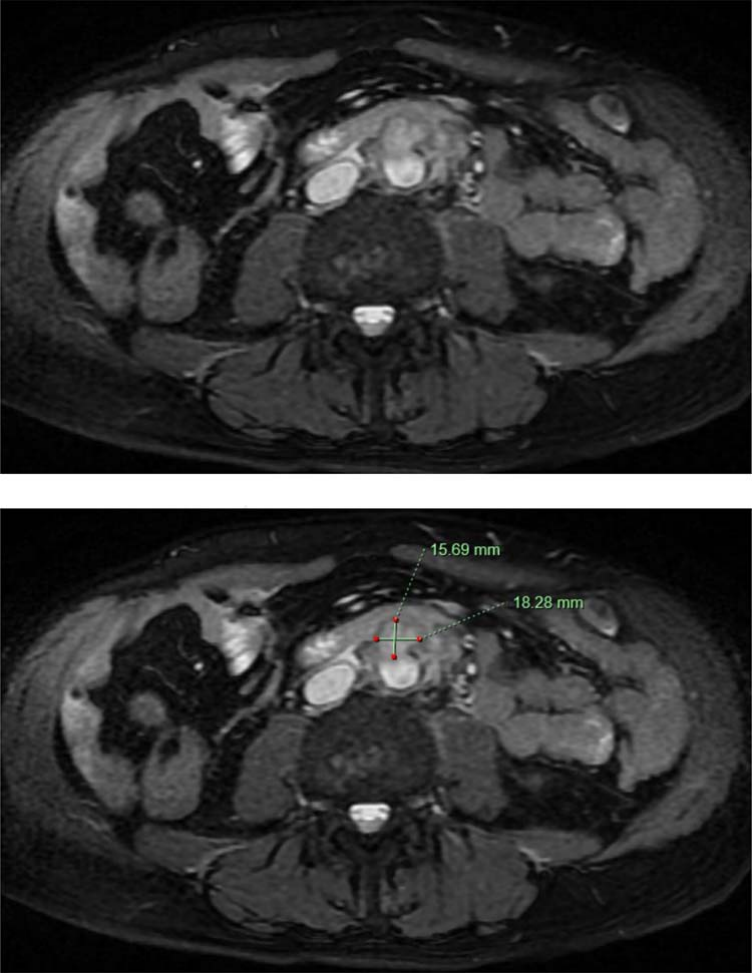
MRCP demonstrating an abdominal aortic saccular aneurysm measuring ~18 × 16mm with associated periaortic soft tissue enhancement suggestive of a mycotic aneurysm. The study was negative for signs of cholangitis.

He was afebrile and had generalized abdominal tenderness, worse over the right upper quadrant, without signs of guarding or peritonism. Murphy’s sign was negative. The provisional diagnosis was progression of his PSC, prompting an urgent magnetic resonance cholangiopancreatogram (MRCP). MRCP revealed an infrarenal saccular aneurysm measuring 18 × 16 mm with associated periaortic soft tissue enhancement. There was no evidence of cholangitis (Fig. 1). Blood culture samples and serology for Q fever, mycoplasma and psittacosis were obtained before commencement of IV ceftriaxone and vancomycin.

**Figure 2:**
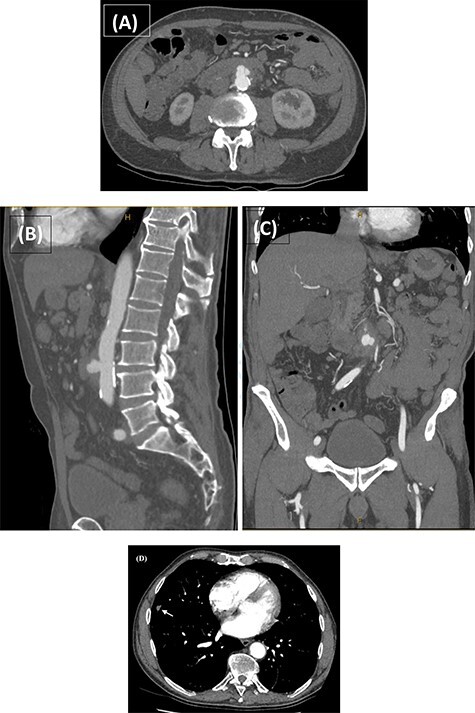
CTA performed following the MRCP demonstrating the presence of a thick-walled multi-lobulated saccular aneurysm arising from the anterior wall of the infrarenal abdominal aorta approximately at the level of the IMA origin. The aneurysm measures ~35 × 23 × 32 mm with adjacent soft tissue stranding and enlarged nodes in the aortocaval and left para-aortic region (**A**–**C**). The incidentally noted right middle lobe nodule is also demonstrated (D; white arrow).

Computed tomography angiography (CTA) confirmed the presence of an infrarenal saccular aneurysm measuring ~35 × 23 × 32mm. Additionally, a right middle lobe pulmonary nodule was noted (Fig. 2), which was further characterized with a high-resolution CT (HRCT) chest. Blood, faecal and urine cultures were negative. Management involved a combination of antibiotic therapy and semi-urgent surgical excision and reconstruction of the infected aorta. Bilateral leg duplex ultrasounds (US) were performed for preoperative planning of an autologous interposition graft using femoral vein (FV), demonstrating no deep venous thrombosis (DVT) and an adequately sized FV measuring 13 mm in diameter.

**Figure 3:**
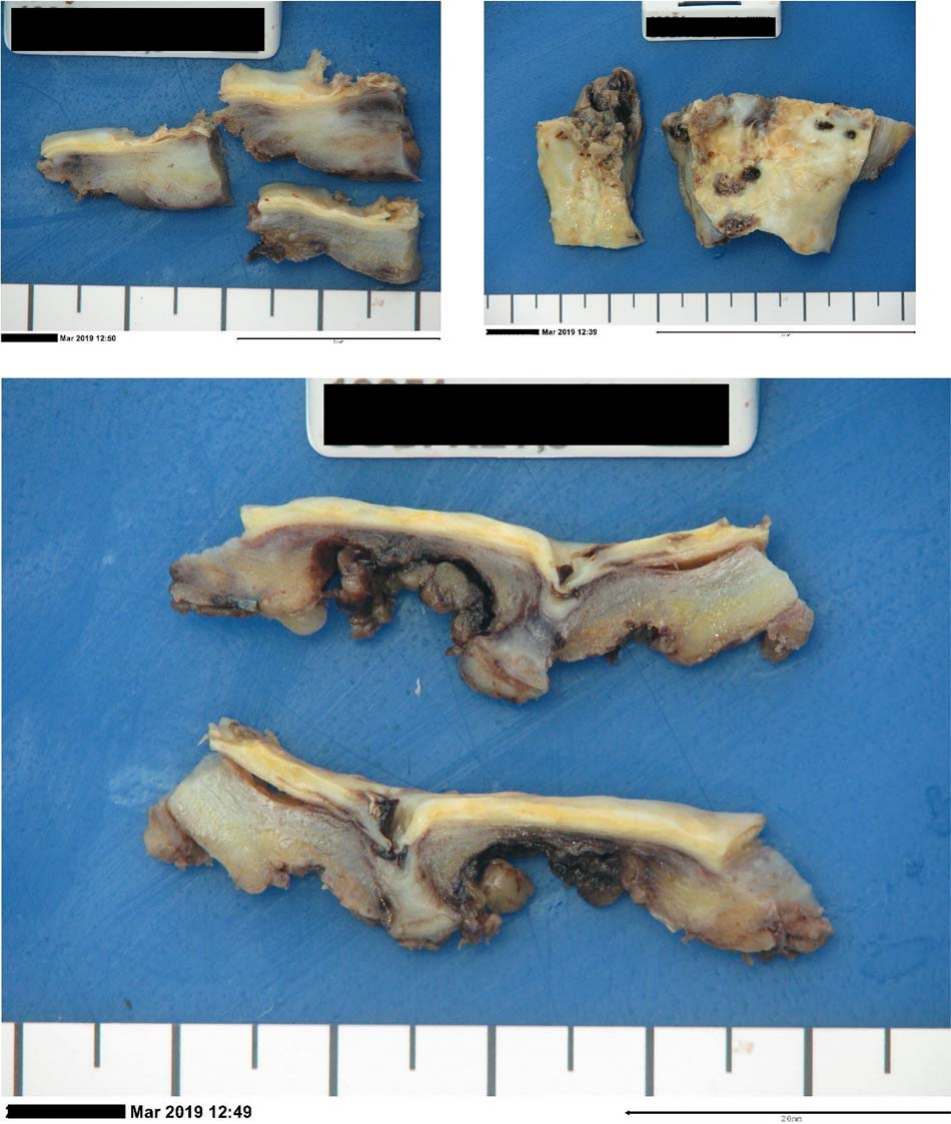
Post-operative laboratory photographs of the resected infra-renal aorta sent for tissue microscopy, culture and sensitivity, as well as histopathological review.

Intraoperatively, dense inflammatory tissue at the mid-infrarenal aorta involving the fourth part of the duodenum, inferior vena cava (IVC), inferior mesenteric artery (IMA) and left accessory lower lobe renal artery (RA) were encountered. After proximal and distal control, the IMA, left accessory RA and lumbar arteries were ligated and the infrarenal aorta was excised. An end-to-end anastomosis with 4-0 prolene was performed in quadrants using the right superficial FV, ensuring alignment. To address the anticipated size discrepancy, the proximal aortic wall was gradually plicated, achieving the desired tapering effect. Clamp time was 58 min. Intraoperative samples were collected (Fig. 3).

The cultured aortic tissue returned positive for *S. equi*, susceptible to penicillin and erythromycin. Unfortunately, subspecies of *S. equi* were not isolated. Following inpatient rehabilitation, he was discharged on post-operative Day 13 on IV ceftriaxone 2 g q24h for 6 weeks.

He continued prophylactic enoxaparin for 30 days and wore thigh high class II compression stockings for prevention of right lower limb complications following FV harvesting. On 30 day follow-up he had made an excellent recovery, returning to his usual activities of daily living. 3-, 6-, 12-, 24- and 30-months follow-up demonstrated a patent aortic autograft, with no evidence of anastomotic stenosis or pseudoaneurysms. HRCT chest showed size reduction of the pulmonary nodules, further supporting an inflammatory process.

## DISCUSSION

Mycotic aneurysms are a rare, accounting for <1% of arterial aneurysms [1]. Generally caused by species with a high affinity for the arterial wall, *S. equi (SE)* mycotic aneurysms are particularly rare [3]. A zoonotic pathogen with three subspecies—*SE equi*, *SE ruminatorum* and *SE zooepidemicus*—SE *zooepidemicus* has been most reported to cause human infections, presenting as bacteremia, meningitis, endocarditis and aortitis [4, 6]. A ß-hemolytic Lancefield group C streptococcal bacterium, *S. equi*, commonly causes infections in horses, pigs, cats and goats. Most reported cases of *S. equi* infection have been linked to contact with infected horses or consumption of unpasteurized milk [6]. The route of transmission in this case was presumed either via droplet transmission or direct contact with an infectious horse.

Patients with mycotic aneurysms present with non-specific constitutional symptoms such as fevers and abdominal or back pain, complicating early diagnosis. A high index of suspicion should be maintained in immunocompromised patients (diabetics, renal failure, chemotherapy, immunosuppressed or malnourished) who have had exposure to causative pathogens such as in our case [3].

Management of mycotic aneurysms involves a combination of surgical resection of the infected tissue for source control and to minimize the high risk of rupture that accompanies mycotic aneurysms. Surgical approaches include resection of the infected tissue and anatomic grafting with grafts extra-anatomically or through an uninfected plane, using autogenous vein or prosthetic material. While extra-anatomical bypass with aortic stump ligation was considered in this case, given the patients premorbid function, we opted for the optimal solution of *in situ* reconstruction given the high long-term durability, low rate of early occlusion (<4%), long-term patency (5-year primary patency rate 75 to 91%) and rarity of recurrent infection (<2%) associated with *in situ* reconstruction utilizing autogenous vein. Additionally, by completely resecting the infected tissue and utilizing autogenous vein as a conduit, we reduced the need for long-term antibiotic therapy that would be required with the use of prosthetic bypass grafting for infected aneurysms, another important consideration for our patient [7].

Generally, broad-spectrum cover is initiated until the causative organism is isolated [3, 8]. The available literature recommends a minimum of 6 weeks IV antibiotic therapy followed by long-term oral antibiotics; however, no definitive guidelines exist [9].

*S. equi* is a virulent bacterium with high morbidity and mortality if left untreated [10]. Awareness of its potential to cause mycotic aneurysms is paramount to diagnosis, particularly if the appropriate risk factors such as exposure to horses are evident.

## References

[1] Reddy DJ, Shepard AD, Evans JR, Wright DJ, Smith RF, Ernst CB. Management of infected aortoiliac aneurysms. Arch Surg 1991;126:873–8 discussion 878-879. 1854247 10.1001/archsurg.1991.01410310083012

[2] Osler W. The Gulstonian Lectures, on Malignant Endocarditis. Br Med J 1885;1:467–70. 10.1136/bmj.1.1262.467 PMC2255866 20751186

[3] Kim Y-W. Infected aneurysm: current management. Ann Vasc Dis 2010;3:7–15. 23555382 10.3400/avd.AVDctiia09003 PMC3595820

[4] Madani A, Zeebregts CJ, Lamprou A, Tielliu IFJ. Mycotic Abdominal Aortic and Iliac Aneurysm Caused by Streptococcus equi Subspecies zooepidemicus Bacteremia. Aorta (Stamford) 2019;7:172–5. 32074645 10.1055/s-0039-3401995 PMC7145433

[5] Matsubayashi Y, Takashima N, Kondo Y, Wakisaka H, Suzuki T. Infected Aortic Aneurysm Caused by Streptococcus zooepidemicus: A Case Report and Literature Review. Ann Vasc Dis 2021;14:71–4. 33786105 10.3400/avd.cr.20-00133 PMC7991697

[6] Trell K, Nilson B, Petersson AC, Rasmussen M. Clinical and microbiological features of bacteremia with Streptococcus equi. Diagn Microbiol Infect Dis 2017;87:196–8. 27829495 10.1016/j.diagmicrobio.2016.10.018

[7] Chung J, Clagett GP. Neoaortoiliac System (NAIS) procedure for the treatment of the infected aortic graft. Semin Vasc Surg 2011;24:220–6. 22230677 10.1053/j.semvascsurg.2011.10.012

[8] Leon LR, Ihnat DM, Mills JL. chapter 41 - Native Arterial Infections. In: Hallett JW, Mills JL, Earnshaw JJ, Reekers JA, Rooke TW (eds). Comprehensive Vascular and Endovascular Surgery, Second edn. Philadelphia: Mosby, 2009, 714–26.

[9] Brown SL, Busuttil RW, Baker JD, Machleder HI, Moore WS, Barker WF. Bacteriologic and surgical determinants of survival in patients with mycotic aneurysms. J Vasc Surg 1984;1:541–7. 6436514

[10] Wilson Walter R, Bower Thomas C, Creager Mark A, et al. Vascular Graft Infections, Mycotic Aneurysms, and Endovascular Infections: A Scientific Statement From the American Heart Association. Circulation 2016;134:e412–60. 27737955 10.1161/CIR.0000000000000457

